# Association of semaglutide use with outcomes in chronic plantar heel pain: a prospective observational cohort and a pilot interventional study

**DOI:** 10.1097/JS9.0000000000003156

**Published:** 2025-08-08

**Authors:** Fan Yang, Lenian Zhou, Jieyuan Zhang, Qiuke Wang, Qianying Cai, Jiazheng Wang, Chenglin Wu, Xueqian Li, Jinshan Zhang, Yongqiang Zheng, Xin Ma, Hongyi Zhu, Zhongmin Shi

**Affiliations:** a Department of Orthopaedics, Shanghai Sixth People’s Hospital, Shanghai, People’s Republic of China; b Institute of Clinical Research, National Center for Orthopaedics, Shanghai Sixth People’s Hospital, Shanghai, People’s Republic of China; c Department of Orthopaedics, Huashan Hospital, Fudan University, Shanghai, People’s Republic of China; d Department of Orthopaedics, Jinjiang Municipal Hospital, Fujian, People’s Republic of China

**Keywords:** GLP1-RA, obesity, plantar fasciitis, plantar heel pain, semaglutide, weight loss

## Abstract

**Background:**

Whether patients with chronic plantar heel pain (PHP) can benefit from glucagon-like peptide-1 receptor agonists (GLP1-RAs) remained unclear.

**Methods:**

This study included a prospective observational cohort and a pilot interventional component. In the observational arm, more than 3000 adults with chronic PHP (duration of symptoms >6 months) were recruited with at least 2-year follow-up from two medical centers. The primary endpoint was the change from baseline of Foot Health Status Questionnaire (FHSQ) pain subdomain at the last follow-up. Secondary endpoints included first-step pain, FHSQ function subdomain, the amount of NSAIDs consumption, number of injection therapies/extracorporeal shockwave therapy (ESWT) sessions/manual stretching, days of orthosis wearing and thickness of plantar fascia. As supporting evidence, a pilot interventional study was conducted in 30 patients with chronic PHP who received semaglutide, with outcomes assessed over 12 months using before-and-after evaluations of pain, function, and imaging.

**Results:**

In the prospective observational cohort, 92 out of 2902 patients who received semaglutide in purpose of treating type 2 diabetes (T2DM) and/or weight loss in the final analysis were identified. Change from baseline in FHSQ pain subdomain was significantly higher in semaglutide group compared with control group (adjusted mean difference, 14.86 [95% CI, 9.97 to 19.75], *P* < 0.001), favoring semaglutide. Similar results were observed from the analysis of FHSQ function subdomain, first-step pain and several therapeutic interventions (oral and topical NSAIDs, corticosteroid injection and orthosis wearing). For structural alterations, plantar fascia thickening velocity was significantly lower in the semaglutide group when compared with control group (−0.25 mm/year [−0.41 to −0.09], *P* = 0.002). For interventional use of semaglutide, improvements in all measured outcomes (FHSQ pain/function subscales, first-step pain and thickness of plantar fascia) were observed with all *P* values less than 0.001. In the interventional substudy, mean FHSQ pain scores improved from 28.09 ± 10.34 at baseline to 60.28 ± 21.34 at follow-up (*P* < 0.001). FHSQ function subdomain scores increased from 27.23 ± 17.44 to 80.71 ± 19.55 (*P* < 0.001).

**Conclusions:**

Semaglutide might be a potential therapeutic candidate for chronic PHP patients by improving patient-reported outcomes and plantar fascia thickening. Further randomized trial is warranted by our study to further evaluate the therapeutic effects of GLP-1RAs on chronic PHP.

## Introduction

Plantar heel pain (PHP) is a medical condition characterized by first-step pain after a period of inactivity and pain during/after weight-bearing activities, affecting approximately 5-7% of the general population.^[1–3]^ PHP lifetime prevalence estimates of PHP are even up to 34.7% in the general population^[4]^. Chronic plantar heel pain has a significant negative impact on general health-related quality of life, independent of age, sex, or body mass index (BMI)^[5]^. The major risk factors of PHP factors can be classified as intrinsic factors (e.g., anatomy features of foot and ankle, gastrocnemius muscle tightness and age) and excessive load from various sources (e.g., overweight/obesity, excessive running, prolonged standing).^[6–9]^ Notably, a range of terms have been also used to describe PHP including plantar fasciitis, jogger’s heel, plantar fasciopathy, and plantar fasciosis^[10]^.

Commonly, PHP is a self-limiting condition with expectation on complete resolution of symptoms within three months^[11]^. Acute PHP may evolve into a chronic condition (duration of symptoms >6 months), with persistent pain, functional disability and impaired life quality^[2,12]^. The major structural alterations of chronic PHP can been observed in the plantar fascia, a dense connective tissue from the metatarsal heads to the medial tubercle of the calcaneus^[13]^. Traditionally viewed as a localized degenerative condition of the plantar fascia due to mechanical overload, recent studies suggest contributions from systemic metabolic and inflammatory factors. Obesity, insulin resistance, and low-grade systemic inflammation may alter connective tissue biology and exacerbate chronic pain states^[12,14,15]^.

First-line therapies such as stretching, orthotics, NSAIDs, corticosteroid injections, and extracorporeal shockwave therapy are widely used, yet many patients fail to achieve durable relief. There is a lack of pharmacologic interventions that target both the mechanical and metabolic contributors to PHP. Despite many previous studies have repeatedly confirmed the association between body weight and PHP, only one previous study, to the best of our knowledge, revealed that weight loss after bariatric surgery could significantly decrease the number of visits for PHP and contribute to symptomatic improvement^[16]^. Because body weight is a modifiable factor, weight loss in nature carries great potential in management on chronic PHP. Traditionally, dietary restriction and physical exercise are the primary options for weight management.^[17–19]^

Glucagon-like peptide-1 receptor agonists (GLP-1RAs) including semaglutide and tirzepatide are effective and safe options for type 2 diabetes (T2DM) and weight control by stimulating delaying gastric emptying and decreasing appetite.^[20–23]^ In addition, GLP-1RAs also have anti-inflammatory effects in direct and indirect fashion^[24,25]^. Given the multifactorial etiology of PHP – particularly in overweight populations – the dual action of semaglutide may offer a novel approach to managing this condition. Its ability to reduce mechanical load through weight loss and its emerging anti-inflammatory and tissue-modifying effects raise the possibility of both symptomatic and structural benefits. To our knowledge, no studies have investigated the effects of semaglutide on pain, function, or plantar fascia remodeling in PHP. This study aims to address that gap through a prospective observational analysis and exploratory intervention.

## Methods

### Study design

This study comprised a prospective observational cohort and a pilot interventional component. Ethical approval was obtained from the institutional review board, and written informed consent was secured from all participants prior to enrollment. This study complied with the Declaration of Helsinki for research. We performed analysis and reported the findings according to the Strengthening the Reporting of Cohort, Cross-sectional, and Case-control Studies in Surgery (STROCSS) criteria^[26,27]^.

**HIGHLIGHTS**

- Overweight/obesity is associated with chronic PHP.
- Results of this observational study indicated effects of GLP-1RAs on PHP progression.
- Semaglutide might be potentially disease-modifying drugs for PHP.

### Participants for prospective observational cohort

From 1 January 2020 to 1 January 2022, we enrolled a total of 3446 patients with chronic PHP (duration of symptoms >6 months) and aged >18 years old in Shanghai Sixth People’s Hospital and Jinjiang Municipal Hospital. Participants were excluded from the final analysis if they had GLP-1RA exposure before enrollment (n = 19), receiving other GLP-1RAs during study period (n = 19), follow-up less than 2 years (n = 267), bilateral plantar fasciitis (n = 144), autoimmune diseases (n = 13), infections or tumors of the lower extremity (n = 33), neurological or vascular abnormality affecting the lower extremity (n = 12) and surgery history of lower extremity at baseline (n = 37) (Supplemental Digital Content Figure S1, available at: http://links.lww.com/JS9/E862). The clinical diagnosis of PHP was all made by specialists in orthopedic surgery and/or sports medicine. The diagnosis of PHP was determined based on pain and tenderness at the inferior heel which was most severe on weightbearing after periods of rest and following prolonged weightbearing. In this study, patients were grouped by receiving semaglutide or not in purpose of treating T2DM and weight management. Notably, semaglutide was not commercially available until early 2022 in China^[28,29]^.

### Baseline data collection

Demographic features at baseline including sex, age, occupational information and education were self-reported by the participants. Height and weight were measured and recorded by researchers. A standard lateral radiograph of the calcaneus was performed for all patients at baseline to grade the plantar heel spurs according to a previous study^[30]^. The size of plantar heels spurs was obtained by averaging the readouts from three radiologists specialized in musculoskeletal radiology. The size of plantar heel spurs was classified as <5 mm, 5–10 mm and >10 mm^[30]^. Daily weightbearing hours were self-reported by patients according to their experiences of the past 7 days.

### Follow-up and patient-reported outcomes

Participants were routinely followed up once per year by phone call and/or WeChat, a popular free messaging app in China. For patient-reported outcomes (PROs), the Foot Health Status Questionnaire (FHSQ) was used at baseline and subsequent annual follow-up^[31]^. The FHSQ, a validated patient-reported outcome instrument, is a 13-item questionnaire used with four subdomains (pain, function, footwear, and general foot health) on a 100-point scale for each subdomain (higher scores represent better foot outcomes). The pain and function subdomains exhibit excellent internal consistency, high test-retest reliability, and established construct validity when compared to other foot-specific and general health measures. The minimal clinically important difference (MCID) has been determined as 12.5 and 7 points for the pain and function subdomains respectively in patients with PHP^[32,33]^. First-step pain was measured on a 100 mm visual analogue scale (VAS), higher score indicating more severe pain^[34]^. The MCID for first-step pain on VAS was −19 mm out of 100 mm for PHP patients^[32,33]^. The details on receiving several common therapeutic interventions including extracorporeal shockwave therapy (ESWT), manual stretching, corticosteroid injections and orthoses were also reported by patients^[2]^. The number of manual stretching, ESWT and corticosteroid injection were reported by patients initially and then confirmed by their medical records. The use of orthoses was reported and recorded based on the number of days used in the past month by the recalling of participants.

### Consumption of non-steroidal anti-inflammatory drugs (NSAIDs) measured by recommended daily dose (RDD)

Both oral and topical NSAIDs were widely used for symptom relieving in patients with PHP^[35]^. We standardized the consumption of different NSAIDs according to a previous study^[28]^. We converted the quantities of NSAID consumption into their RDDs. The RDD was defined as the recommended daily dose given by its labeling information. If multiple recommended doses existed, the RDD was calculated by averaging the highest and lowest recommended doses. Details of RDD were summarized in Supplemental Digital Content Table S1 (available at: http://links.lww.com/JS9/E862).

### Measurement of plantar fascia thickness on MRI

The plantar fascia thickness measured on MRI was positively associated with age and the severity of chronic PHP according to several previous studies.^[36–38]^ Reduction in plantar fascia was often considered as a sign of disease remission by many clinical trials^[39–41]^. We did not perform scheduled MRI scans for each participant of this study. MRI scans in this study were performed by the patient preference and recommendation from his or her treating physician. For the comparison between semaglutide and control groups, we collected the earliest and latest MRI scans during the study period. We only included those patients who received at least two MRI scans with a minimal 6-month interval. We measured the thickness of plantar fascia on the sagittal section at 1 cm away from its calcaneal origin on proton density weighted sequences by the methods reported previously^[12,36]^.

### Preliminary study for interventional use of semaglutide in treating chronic PHP

Based on the findings from the observational cohort study, we hypothesized that semaglutide may be a potential therapeutic option for patients with chronic plantar heel pain (PHP). To further evaluate this hypothesis, an additional 30 patients with chronic PHP were recruited between January 2023 and January 2024, using the same inclusion and exclusion criteria as the observational cohort. Participants received once-weekly semaglutide at a dose of 1.0 mg. A before-and-after comparison was conducted over 12 months, with assessments including the FHSQ pain and function subscales, first-step pain, and plantar fascia thickness measured by MRI at baseline and at 1-year follow-up.

### Statistical analysis

**Figure 1.**
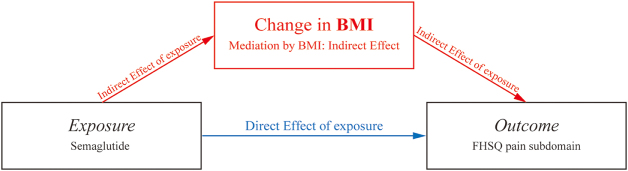
Directed acyclic graph for mediation relationships.

Continuous and categorical variables are presented as means ± SD and counts (percentages), unless otherwise indicated. Univariable analyses were first performed via the t-test (or Mann-Whitney U test) and Pearson’s χ2 test (or Fisher’s exact test). Multivariable linear regression models were used to compare the mean difference in change from baseline for FHSQ and first-step pain after adjusting for age, sex, BMI, and baseline pain scores) or any other covariates with a *P* value less than 0.2 yielded by the univariable analysis (T2DM and smoking). We also established an exposure-mediator-outcome model to assess weight loss and glycemic control as a mediator. In this model, the total effect of semaglutide exposure on the outcomes was divided into the direct and indirect effects. We performed a model-based causal mediation analysis to calculate the proportion of indirect effect and its 95% CI (simulated by the quasi-Bayesian Monte Carlo method based on normal approximation)^[42]^. Missing data were initially assessed for patterns and proportions. For primary analyses, a complete-case approach was applied. When the proportion of missing data for any given outcome exceeded 5%, multiple imputation using chained equations (MICE) was conducted under the assumption that data were missing at random (MAR). Imputation models included all variables used in the final analyses to preserve consistency and reduce bias. The set of pre-exposure covariates (age, sex, BMI and baseline FHSQ pain scores) satisfied the assumption of confounding adjustment for the exposure–mediator–outcome relationships (Fig. 1).

Several sensitivity analyses were conducted. First, we conducted on a responder analysis to comparing the proportion of patients within the two groups who experienced an improvement greater than the MCIDs of FHSQ pain/function subdomains. This method can avoid the possibility that the favoring mean difference was skewed by outliers in such and most patients did not have an effect greater than the MCID^[43]^. Second, to prevent the occurrence of a possible ceiling effect where increase in FHSQ function domain might be difficult to discern, we excluded patients with FHSQ function scores greater than 93 at baseline (MCID = 7). No case had a FHSQ pain score greater than 87.5 at baseline (MCID = 12.5). A post hoc power calculation was performed using the primary outcome measure (change in FHSQ pain score), the study had >80% power to detect a mean difference of at least 12.5 points.

All statistical assessments were performed in a two-sided fashion. When *P* value was less than 0.05, the result would be considered as statistically significant. Statistical analysis was conducted using IBM SPSS V.26.0, and the “mediation” package in R V.4.1.2 was applied for mediation analysis in this study.

## Results

### Body weight and change from baseline in the semaglutide and control groups

A total of 2902 patients were included for the final analysis and we identified 92 participants who received semaglutide in purpose of treating T2DM (n = 67) and/or weight loss (n = 76). The baseline characteristics of semaglutide and control groups were demonstrated in Table 1. The semaglutide and control groups had similar mean BMI at baseline (26.67 ± 3.67 versus 26.05 ± 3.99, *P* = 0.141). At the last follow-up, the semaglutide group had a significantly lower change from baseline in BMI compared with the control group (semaglutide versus control, −2.14 ± 3.14 versus 0.83 ± 2.00, adjusted mean difference −2.89 [−3.33 to −2.45], *P* < 0.001) (Table 2).

**Table 1:** Baseline characteristics of patients grouped by semaglutide exposure

	Semaglutide (n = 92)	Control (n = 2810)	*P* value
Age, years	51.28 ± 7.97	50.44 ± 8.63	0.358
Sex, No. (%)			
Male	43 (46.7)	1137 (40.5)	0.228
Female	49 (53.3)	1673 (59.5)	
BMI, kg/m^2^	26.67 ± 3.67	26.05 ± 3.99	0.141
Duration since the first PHP episode, months	39.84 ± 18.93	38.81 ± 19.17	0.616
Daily weightbearing, hours	3.45 ± 2.60	3.48 ± 2.42	0.908
Hypertension, No. (%)			
Yes	35 (38.0)	1062 (37.8)	0.961
No	57 (62.0)	1748 (62.2)	
Smoking			
Yes	6 (6.5)	315 (11.2)	0.158
No	86 (93.5)	2495 (88.8)	
T2DM, No. (%)			
Yes	67 (72.8)	466 (16.6)	<0.001
No	25 (27.2)	2344 (83.4)	
Heel spur size, No. (%)			
<5 mm	65 (70.6)	1841 (65.5)	0.462
5–10 mm	25 (27.2)	850 (30.2)	
>10 mm	2 (2.2)	119 (4.3)	
FHSQ pain subdomain	32.47 ± 20.35	35.06 ± 20.67	0.237
FHSQ function subdomain	57.13 ± 21.86	59.64 ± 21.84	0.280
First-step VAS pain, mm	49.16 ± 20.90	50.26 ± 20.02	0.607

Data are shown as means (± SD) unless otherwise indicated. BMI, body mass index; T2DM, type 2 diabetes; FHSQ, Foot Health Status Questionnaire; VAS, visual analogue scale.

**Table 2:** Comparison of PROs and therapeutic strength between semaglutide and control groups

	Semaglutide (n = 92)	Control (n = 2810)	Adjusted mean difference (95% CI) *	Adjusted *P* value*
Follow-up, years	2.89 ± 0.69	3.03 ± 0.69		
BMI, kg/m^2^				
At last follow-up	24.54 ± 4.62	26.88 ± 4.46		
Change from baseline	−2.14 ± 3.14	0.83 ± 2.00	−2.90 (−3.34, −2.45)	<0.001
FHSQ pain subdomain				
At last follow-up	60.67 ± 23.74	48.14 ± 27.38		
Change from baseline	28.19 ± 24.65	13.08 ± 23.34	14.86 (9.97, 19.75)	<0.001
FHSQ function subdomain				
At last follow-up	79.35 ± 19.36	69.06 ± 24.42		
Change from baseline	22.21 ± 21.46	9.43 ± 21.56	12.22 (7.91,16.54)	<0.001
First-step VAS pain, mm				
At last follow-up	27.46 ± 15.67	35.51 ± 19.11		
Change from baseline	−21.71 ± 23.54	−14.75 ± 27.46	−6.27 (−12.15, −0.39)	0.037
Consumption of oral NSAIDs in last year, RDDs	13.74 ± 18.08	17.50 ± 11.36	−4.10 (−6.61, −1.60)	0.001
Consumption of topical NSAIDs in last year, RDDs	35.53 ± 47.85	69.47 ± 74.21	−31.79 (−47.61, −15.98)	<0.001
Number of corticosteroid injections in last year	0.08 ± 0.27	0.20 ± 0.48	−0.14 (−0.24, −0.04)	0.008
Number of ESWT sessions in last year	0.70 ± 1.40	1.05 ± 2.08	−0.38 (−0.82, 0.07)	0.095
Number of manual stretching in last year	1.02 ± 1.37	1.26 ± 1.79	−0.25 (−0.63, 0.14)	0.209
Days of wearing orthoses in last month	3.65 ± 6.52	7.18 ± 9.16	−3.15 (−5.10, −1.20)	0.002

Data are shown as means (± SD) unless otherwise indicated. BMI, body mass index; NSAIDs, nonsteroidal anti-inflammatory Drugs; RDD, recommended daily dose; ESWT, extracorporeal shock wave therapy; FHSQ, Foot Health Status Questionnaire; VAS, visual analogue scale.

^*^ Mean difference and *P* value adjusted for age, sex, baseline BMI, smoking, T2DM and baseline FHSQ pain subdomain.

### Comparison of PROs between the semaglutide and control groups

As shown in Table 2, after controlling baseline characteristics including age, sex, baseline BMI, smoking, T2DM and baseline FHSQ pain subdomain, the adjusted analysis of FHSQ pain subdomain showed higher change from baseline in the semaglutide group than in the control group at last follow-up, with an adjusted mean difference of 14.86 (95% confidence interval [CI], 9.97 to 19.75; *P* < 0.001). The analysis of first-step pain showed similar results, with an adjusted mean difference of −6.27 (95% CI, −6.27, −12.15 to −0.39, *P* = 0.037), favoring semaglutide group. For FHSQ function subdomain, patients in the semaglutide group achieved clinically meaningful improvement compared with control group (adjusted mean difference [95% CI], 12.22 [7.91 to 16.54], *P* < 0.001). The association of semaglutide exposure with improvement in FHSQ pain/function subdomain was substantially mediated by the BMI reduction (mediation proportion: FHSQ pain subdomain, 52.6% [36.2% to 79.0%], *P* <0.001; FHSQ function subdomain, 56.6% [39.9% to 82.0%], *P* <0.001) (Table 3 and Fig. 1).

**Table 3:** Direct effect of semaglutide and indirect effect mediated by weight loss

Exposure: semaglutide	Mediator: change in BMI endpoint: change in FHSQ pain subdomain*	*P* value
Controlled direct effect	7.07 (2.11, 12.03)	0.008
Indirect effect	7.76 (5.71, 10.01)	<0.001
Total effect	14.83 (9.40, 19.82)	<0.001
Proportion mediated	52.6% (36.2%, 79.0%)	<0.001
Controlled direct effect	5.33 (1.52, 9.09)	0.008
Indirect effect	6.88 (5.07, 8.88)	<0.001
Total effect	12.21 (7.88, 16.21)	<0.001
Proportion mediated	56.6% (39.9%, 82.0%)	<0.001

FHSQ, Foot Health Status Questionnaire.

* The model was adjusted for age, sex, baseline BMI, smoking, T2DM and baseline FHSQ pain subdomain.

For sensitivity analysis, we compared the proportions of patients who achieved MCIDs of FHSQ pain/function subdomains between the semaglutide and control groups (pain: 71.7% versus 52.8%, *P* < 0.001; function: 69.6% versus 49.0%, *P* < 0.001; both: 59.8% versus 45.2%, *P* = 0.006) (Supplemental Digital Content Table S2, available at: http://links.lww.com/JS9/E862). Additionally, analyses of PROs after excluding patients with too greater baseline FHSQ function scores (>93) also showed stable and consistent results (Supplemental Digital Content Table S3, available at: http://links.lww.com/JS9/E86).

### Comparison of therapeutic interventions between the semaglutide and control groups

As shown in Table 2, we observed statistically significant decrease in consumption of oral NSAIDs (adjusted mean difference [95% CI], −4.10 [−6.61 to −1.60] RDDs, *P* = 0.001), topical NSAIDs (−31.79 [−47.61 to −15.98] RDDs, *P* < 0.001), number of corticosteroid injections (−0.14 [−0.24 to −0.04], *P* = 0.008) in the year prior to the last follow-up. For ESWT and manual stretching, we did not observe statistically significant difference between the semaglutide and control groups in the year prior to the last follow-up. In the month before the last follow-up, patients in the semaglutide group required fewer days for orthosis bearing compared with the control group (−3.15 [−5.10, −1.20], *P* = 0.002).

### Comparison of structural outcomes between the semaglutide and control groups

In this study, we found 18 and 389 patients who received at least two MRI scans from the semaglutide and control groups during the study period. The mean durations between the two MRI scans for the semaglutide and control groups were 1.98 ± 0.51 and 1.79 ± 0.84 years, with a *P* value of 0.159. After adjusting for baseline characteristics including age, sex, baseline BMI, smoking, T2DM and baseline FHSQ pain subdomain, plantar fascia thickening velocity was significantly lower in the semaglutide group when compared with control group (adjusted mean difference, −0.25 mm/year [95%CI, −0.41 to −0.09], *P* = 0.002) (Table 4).

**Table 4:** Comparison of structural changes between semaglutide and control groups

	Semaglutide (n = 18)	Control (n = 389)	*P* value
Interval between MRI scans, years	1.98 ± 0.51	1.79 ± 0.84	0.159
Change in thickness of plantar fascia, mm	−0.08 ± 1.14	0.30 ± 0.59	0.027*
Plantar fascia thickening velocity, mm/year	−0.07 ± 0.54	0.18 ± 0.29	0.002*

* *P* values were adjusted for age, sex, baseline BMI, smoking, T2DM and baseline FHSQ pain subdomain.

### Interventional use of semaglutide in treating chronic PHP

After observational phase, we further recruited 30 patients with chronic PHP with the identical inclusion and exclusion criteria of the observational study. We then conducted a before-and-after comparison study of these 30 patients. All patients completed the 1-year follow-up including patient-reported outcomes and MRI-based thickness measurement of plantar fascia thickness. In this exploratory phase, 30 patients with chronic PHP were treated with semaglutide and followed for 12 months. All completed both baseline and follow-up assessments. Mean FHSQ pain scores improved from 28.09 ± 10.34 at baseline to 60.28 ± 21.34 at follow-up (*P* < 0.001). FHSQ function subdomain scores increased from 27.23 ± 17.44 to 80.71 ± 19.55 (*P* < 0.001). First-step pain on a 100-mm VAS decreased from 40.28 ± 11.79 mm to 8.76 ± 8.61 mm (*P* < 0.001). MRI-measured plantar fascia thickness declined from 5.38 ± 1.05 mm to 4.20 ± 0.59 mm (*P* < 0.001) (Table 5). Additionally, no serious adverse events were reported during the follow-up period in the interventional cohort. The most commonly reported side effects were gastrointestinal in nature, including transient nausea (5 patients), reduced appetite (3 patients), and mild constipation (2 patients). All symptoms were self-limited and did not lead to discontinuation of semaglutide therapy. No hypoglycemic episodes, pancreatitis, or other systemic complications were observed.

**Table 5:** Before-and-after comparison of PROs and structural changes in patients with interventional semaglutide use

	Baseline (n = 30)	One-year follow-up (n = 30)	*P* value*
BMI, kg/m^2^	25.54 ± 4.72	21.88 ± 4.18	<0.001
FHSQ pain subdomain	28.09 ± 10.34	60.28 ± 21.34	<0.001
FHSQ function subdomain	27.23 ± 17.44	80.71 ± 19.55	<0.001
First-step VAS pain, mm	40.28 ± 11.79	8.76 ± 8.61	<0.001
Thickness of plantar fascia, mm	5.38 ± 1.05	4.20 ± 0.59	<0.001

Data are shown as means (± SD) unless otherwise indicated. BMI, body mass index; FHSQ, Foot Health Status Questionnaire; VAS, visual analogue scale.

## Discussion

This is the first clinical investigation to explore the potential effects of semaglutide on chronic PHP. After semaglutide usage, the FHSQ pain/function subdomain and first-step pain consistently improved when compared with the control group. Accompanied with the improvement on PROs, we also observed a significant decrease in several therapeutic interventions including oral/topical NSAIDs, corticosteroid injection and orthosis wearing after using semaglutide. Finally, plantar fascia thickening velocity was significantly lower in the semaglutide group when compared with control group. The lower limit of the 95% CI (LLCI) of adjusted mean difference in FHSQ function subdomain (7.91 points) was higher than the MCID (7 points). The point estimate of adjusted mean difference in FHSQ pain subdomain (14.86 points) was higher than the MCID (12.5 points) while the LLCI (9.97 points) was lower than the MCID (12.5 point). For first-step pain, the point estimate of adjusted mean difference was −6.27 mm, much lower than its MCID (−19 mm). Although the point estimate of improvement in the FHSQ pain subdomain exceeded the minimal clinically important difference (MCID) of 12.5 points, the lower bound of the 95% confidence interval (9.97) fell below this threshold. This suggests that while the result is statistically significant, it may not consistently achieve clinically meaningful benefit across the broader patient population. As such, these findings should be interpreted as suggestive but not definitive evidence of meaningful pain improvement. For structural alterations, although we observed a statistically significant slowing of plantar fascia thickening in the semaglutide group (–0.25 mm/year), the clinical relevance of this change remains unclear. While increased fascia thickness has been associated with chronic plantar heel pain in some imaging studies, current evidence does not support a direct correlation between reduction in thickness and symptom improvement^[44,45]^. Therefore, this structural outcome should be considered exploratory, and further research is needed to clarify whether it reflects true disease modification or has predictive value for clinical outcomes.

Mechanical overload at the plantar fascia insertion plays a vital role in the pathogenesis of PHP^[46]^. Many therapeutic interventions for PHP including weight loss, orthoses and management of athletic and occupational workload had been established and recommended based on this core concept. For weight management, even a modest weight loss can significantly reduce the dynamic plantar loading during walking and weightbearing^[47]^. A previous study revealed that weight loss after bariatric surgery could significantly decrease the number of visits for PHP and contribute to symptomatic improvement^[16]^. Likewise, weightbearing redistribution between the bilateral lower limbs is an effective coping strategy for patients to offload the painful heel and relieve pain^[48]^. Although weight loss seemed to be effective in treating PHP, the efficacies of different weight-reducing strategies including diet, exercise and medications should be evaluated in treating chronic PHP. Because the benefits of semaglutide in PHP may be largely mediated by weight loss, we cannot exclude the possibility that similar improvements would be observed with other effective weight loss therapies. However, due to the low number of patients receiving alternative pharmacologic or surgical weight loss interventions in this dataset, a comparator group was not available. Future research – including target trial emulation or randomized comparisons with other agents – would help determine whether semaglutide exerts unique effects on musculoskeletal outcomes or reflects a class-wide benefit linked to weight reduction. Moreover, the causal mediation analysis suggested that approximately 52.6% of the observed improvement in FHSQ pain scores may be mediated through BMI reduction. However, this finding should be interpreted with caution, as the validity of mediation analysis relies on strong assumptions – including the absence of unmeasured confounding between the exposure, mediator, and outcome – which are difficult to verify in observational studies. As such, the mediation results are exploratory and should be validated in future randomized or mechanistically controlled trials.

The semaglutide was approved by Chinese regulatory agency in 2021 and commercially available in early 2022 and the exposures of other GLP-1RAs were found in less than 0.5% of the general population before 2022^[28,29]^. These facts gave us a unique research opportunity to explore the potential effects of semaglutide on chronic PHP. In this study, we only needed to exclude 19 out of 3446 participants from the final analysis because of GLP-1RA exposure before enrollment, without introducing substantial selection bias. Notably, GLP-1RA therapies also led to a substantial reduction in the lean body due to the hypocaloric dieting^[22,49]^. The calf circumference and muscle strength measured by grip strength did not decrease significantly after GLP-1RA using^[50]^. Considering the potential role of calf tension on PHP, future studies should pay attention to the impact of GLP-1RAs on the tension, mass and strength of calf muscle. Importantly, semaglutide is currently only indicated for patients with type 2 diabetes or individuals with obesity or overweight and associated comorbidities. Its potential application in normal-weight individuals with PHP presents several challenges. First, the majority of symptom improvement observed in our study appears to be mediated by weight loss, which would not be a therapeutic goal in normoweight patients. Second, the off-label use of semaglutide in this group may carry safety risks, including unintended weight loss, nutritional deficiencies, or sarcopenia. Finally, the anti-inflammatory properties of semaglutide, while promising, remain insufficiently characterized to support clinical use independent of its metabolic effects. Thus, our findings should not be extrapolated to patients with normal BMI, and further research is required to explore its role, if any, in this subgroup.

The current study has several limitations. First, although we have adjusted our analysis for T2DM and baseline BMI, indication bias remained inevitable due to its observational nature. Since our preliminary outcomes of interventional use of semaglutide were promising, we would say the conclusion of this study should be reliable. Nevertheless, clearly, future randomized trials are still needed to further validate our findings in this study. This study would not establish any confirmatory conclusions on the efficacy of GLP-1RA therapies on chronic PHP. Second, our conclusions were proposed largely based on the PROs instead of “hard” endpoints. Although we collected the data regarding various therapeutic interventions, none of these treatments could be considered as a milestone event for disease progression^[2]^. Plantar fasciotomy has been recommended for treating chronic PHP while the incidence of this surgery in real world might be very low^[51,52]^. No participant in our study received plantar fasciotomy during the study period. An additional limitation of this study is the absence of data on physical activity levels, dietary behavior, and other concomitant lifestyle interventions, all of which are known to influence both body weight and musculoskeletal pain outcomes. These unmeasured confounders may have contributed to the observed associations and limit the ability to isolate the specific effects of semaglutide. Moreover, because this is an observational study, the dose and duration of semaglutide varied by patient preference, especially in those patients using semaglutide for weight loss. Finally, a notable limitation of our study is the imbalance in group sizes and baseline characteristics – for instance, only 92 patients received semaglutide compared to 2,810 in the control group, and the prevalence of type 2 diabetes mellitus was significantly higher in the semaglutide group (72.8%) than in the control group (16.6%). Besides, the MRI subgroup analysis included only 18 semaglutide-treated patients. The discrepancy reflects the limited use of semaglutide during the study period and may introduce instability in the statistical models, increase the risk of residual confounding, and limit the precision of the effect estimates. Although we applied multivariable adjustment for key baseline characteristics, residual bias cannot be excluded. As for the interventional substudy, several variables in this study were self-reported by patients and thus may be subject to recall bias and social desirability bias, patients may have overestimated or underestimated their pain relief. Although validated tools such as the FHSQ were used to standardize subjective assessments, the potential for self-reporting bias remains a limitation and may have influenced the observed associations.

### Conclusions

In conclusion, semaglutide might be a potential therapeutic candidate for chronic PHP patients by improving patient-reported outcomes and plantar fascia thickening. Further randomized trial is warranted by our study to further evaluate the therapeutic effects of GLP-1RAs on chronic PHP.

## Data Availability

All relevant anonymized patient-level data are available upon reasonable request to the corresponding author.

## Research registration unique identifying number (UIN)

researchregistry10645 (https://researchregistry.knack.com/research-registry#home/registrationdetails/66d0e83324957702e143fe71/).
